# Isolation of Terpenoids from the Stem of *Ficus aurantiaca* Griff and their Effects on Reactive Oxygen Species Production and Chemotactic Activity of Neutrophils

**DOI:** 10.3390/molecules21010009

**Published:** 2016-01-05

**Authors:** Shukranul Mawa, Ibrahim Jantan, Khairana Husain

**Affiliations:** Drug and Herbal Research Centre, Faculty of Pharmacy, Universiti Kebangsaan Malaysia (UKM), Jalan Raja Muda Abdul Aziz, Kuala Lumpur 50300, Malaysia; shuku_76@yahoo.com (S.M.); profibj@gmail.com (I.J.)

**Keywords:** *Ficus aurantiaca* Griff, terpenoids, immunomodulatory, neutrophils, chemotaxis, reactive oxygen species

## Abstract

Three new triterpenoids; namely 28,28,30-trihydroxylupeol (**1**); 3,21,21,26-tetrahydroxy-lanostanoic acid (**2**) and dehydroxybetulinic acid (**3**) and seven known compounds; *i.e.*, taraxerone (**4**); taraxerol (**5**); ethyl palmitate (**6**); herniarin (**7**); stigmasterol (**8**); ursolic acid (**9**) and acetyl ursolic acid (**10**) were isolated from the stem of *Ficus aurantiaca* Griff. The structures of the compounds were established by spectroscopic techniques. The compounds were evaluated for their inhibitory effects on polymorphonuclear leukocyte (PMN) chemotaxis by using the Boyden chamber technique and on human whole blood and neutrophil reactive oxygen species (ROS) production by using a luminol-based chemiluminescence assay. Among the compounds tested, compounds **1**–**4**, **6** and **9** exhibited strong inhibition of PMN migration towards the chemoattractant *N*-formyl-methionyl-leucyl-phenylalanine (fMLP) with IC_50_ values of 6.8; 2.8; 2.5; 4.1; 3.7 and 3.6 μM, respectively, comparable to that of the positive control ibuprofen (6.7 μM). Compounds **2**–**4**, **6**, **7** and **9** exhibited strong inhibition of ROS production of PMNs with IC_50_ values of 0.9; 0.9; 1.3; 1.1; 0.5 and 0.8 μM, respectively, which were lower than that of aspirin (9.4 μM). The bioactive compounds might be potential lead molecules for the development of new immunomodulatory agents to modulate the innate immune response of phagocytes.

## 1. Introduction

Medicinal plant products have long been used in traditional medicines for the treatment of many immunological disorders. Their therapeutic effects may be due to their effects on the immune system [1]. Many herbs such as *Andrographis paniculata*, *Allium sativum*, *Trigonella foenum graecum*, *Pouteria cambodiana*, *Centella asiatica*, *Asparagus racemosus*, *Baliospermum montanum*, *Curcuma longa*, *Panax ginseng*, *Phyllanthus debilis*, *Tinospora cordifolia*, and *Picrorhiza scrophulariiflora* have been reported to be able to modulate the immune system, including both adaptive and innate arms of the immune responses, exhibiting either immunostimulant or immunosuppressive effects [2,3,4]. Immunomodulation by these plant extracts and their active components provide new sources of lead molecules for development of natural immunomodulators for a variety of immunologic diseases. Assessment of the immunological effects of compounds is based on their selective activities on the different components of the immune system. In recent years, there was an increased interest in the search for natural immunomodulators from medicinal plants to substitute conventional therapy as they are considered to possess fewer side effects [5].

*Ficus* (family: Moraceae) is one of the largest genera of angiosperms, with more than 800 species of trees, shrubs, hemi-epiphytes and climbers in the tropics and sub-tropics worldwide [6]. Due to its high economic and nutritional values the genus is a vital hereditary source and also an important component of biodiversity of the rainforest ecosystem. It is also a good source of food for fruit eating animals in tropical area [7]. Chemical investigations of various *Ficus* species have shown the presence of flavonoids, coumarins, alkaloids, steroids, triterpenoids, simple phenols and salicylic acids from *F. benghalensis*, *F. carica*, *F. hirta*, *F. hispida*, *F. microcarpa*, *F. nymphaeifolia*, *F. ruficaulis* and *F. septica* [8,9]. Some of these plants exhibited a wide range of biological activities such as anti-inflammatory [10,11,12], antioxidant, hypolipidemic and hypoglycemic activities [13]. Some *Ficus* species are well known in Asia as medicinal plants and are widely used in folk medicines for the treatment of flu, malaria, tonsillitis, bronchitis and rheumatism [14]. One of the most biologically active species of *Ficus* is *F. carica* which has been reported to have some 21 traditional and current uses in various ethnopharmacological practices [15].

*Ficus aurantiaca* Griff is an evergreen tree that can grow up to 9 m. It is widespread in the lowland forests at Kelantan, Terengganu, Perak, Pahang, Selangor, Melaka and Johor in Malaysia, where it is locally known as “tengkuk biawak” or “akar tengkuk biawak hitam”. Traditionally, the plant parts have been used for the treatment of various ailments including headache, wound and toothache [16]. There is little investigation on the chemical composition and biological properties of *F. aurantiaca*. Our preliminary study on the stem extract of this plant had demonstrated that it inhibited *in vitro* chemotactic migration as well as suppressed the release of reactive oxygen species by granulocytes (unpublished data). The aims of the present study were to isolate and identify the constituents of the stem of *F. aurantiaca* and evaluate their effects on reactive oxygen species (ROS) production and chemotactic activity of neutrophils.

## 2. Results and Discussion

### 2.1. Characterization of the Isolated Compounds

**Figure 1:**
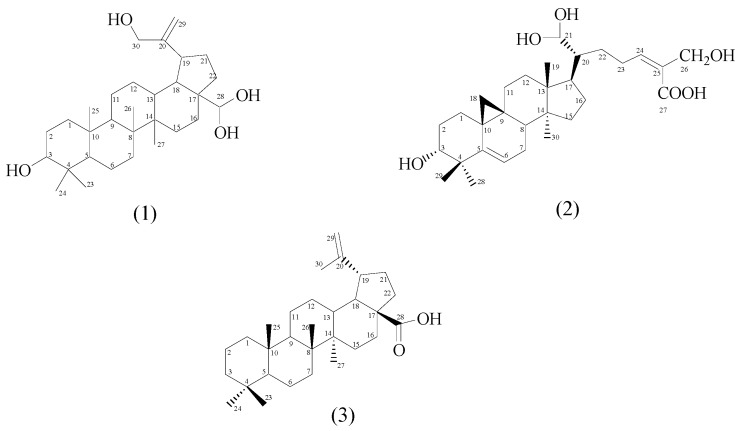
New terpenoids from *F. aurantica*.

Successive separations of *n*-hexane, ethyl acetate and methanol extracts using silica gel chromatography afforded three new triterpenoids, 28,28,30-trihydroxylupeol (**1**), 3,21,21,26-tetrahydroxylanostanoic acid (**2**) and dehydroxybetulinic acid (**3**) along with five known triterpenoids, taraxerone (**4**), taraxerol (**5**), stigmasterol (**8**), ursolic acid (**9**), acetyl ursolic acid (**10**), one sesquiterpenoid, herniarin (**7**) and one diterpenoid, ethyl palmitate (**6**). The structures of the new compounds are shown in Figure 1. The structures of the known compounds were elucidated by the combination of ESIMS, ^1^H- and ^13^C-NMR spectral data and comparison of their spectral data with literature values [17,18,19,20,21,22,23].

Compound **1** (20 mg) was isolated as a white powder with a melting point range of 222–224 °C. The IR spectrum showed a band at 3311 cm^−1^ due to the presence of hydroxyl groups (-OH). C-H stretching vibrations were observed at 2920 cm^−1^ while the methyl bending vibration was at 1411 cm^−1^. The HRESIMS displayed a molecular ion at *m*/*z* 474.1465 [M]^+^, corresponding to a molecular formula of C_30_H_50_O_4_ (M = 474.1465). The ^1^H-NMR spectrum exhibited singlets indicating the presence of five methyl groups, deshielded methine protons at δ_H_ 2.04 (H-18) and 2.38 (H-19) and two olefinic protons at δ_H_ 4.69 and 4.57. The corresponding ^13^C-NMR spectrum displayed 30 carbon signals, including five methyls, twelve methylenes, seven methines and six quaternary carbons. Two methine carbons at δ_C_ 80.1 (C-3) and 95.1 (C-28) were typical for C-OH and two sp^2^ carbons at δ_C_ 150.9 (C-20) and 109.3 (C-29), indicated the presence of one double bond. Another methylene carbon at δ_C_ 69.2 (C-30) was characteristic for hydroxyl (C-OH). The ^1^H- and ^13^C-NMR data of **1** were very similar to those reported for lupeol [24], the main difference being the presence of four hydroxyl groups instead of only one. Two methyl group protons at C-28, were substituted by two hydroxyl groups, and in the C-30 methyl group, a proton was also ubstituted by a hydroxyl group. The positions of the hydroxyl groups were established by the HMQC correlation of the hydroxyl (-OH) protons, δ_H_ 2.37, 2.04, 2.29 and 2.39 with the δ_C_ 80.1 (C-3) 69.2 (C-30) and 95.1 (C-28) carbon signals, respectively.

**Figure 2:**
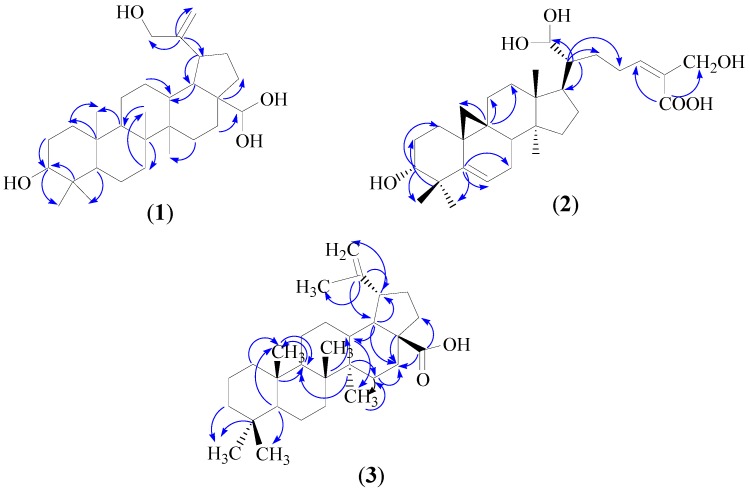
Selected HMBC correlations (C→H) of compounds **1**–**3**.

The connectivity between protons and carbons established by the HMQC and HMBC spectra indicated that the two olefinic protons were attached to carbon at δ_C_ 109.3 (C-29), the two adjacent methyl protons resonating as a singlet at δ_H_ 0.79 (s, 3H, H-23) and 0.83 (s, 3H, H-24) attached to a quaternary carbon at δ_C_ 39.6 (C-4) were attributed to methine carbon δ_C_ 55.6 (C-5) and hydroxyl methine carbon δ_C_ 80.1 (C-3) by ^3^*J* correlation, respectively. Meanwhile another methyl proton at δ_H_ 0.94 (s, 3H, H-25) displayed ^3^*J* correlations with methylene carbon at δ_C_ 38.1 (C-1) and methine carbon at δ_C_ 50.3 (C-9). Another two groups of methyl protons were observed at δ_H_ 1.01 (s, 3H, H-26) and 1.07 (s, 3H, H-27), showed ^3^*J* correlation with methylene carbon at δ_C_ 34.1(C-7) and δ_C_ 27.4 (C-15), respectively. One hydroxyl methine proton at δ_H_ 3.39 (dd, 1H, *J* = 10.8, 4.8 Hz, H-3) showed a ^2^*J* correlation with methylene carbon δ_C_ (C-2). Another three methine protons were visible at δ_H_ 1.34 (m, 1H, H-13), 2.04 (m, 1H, H-18) and 2.38 (d, 1H, *J* = 7.2 Hz, H-19) whereas at δ_H_ 1.34 (m, 1H, H-13) showed ^2^*J* correlation with methylene carbon at δ_C_ 25.1 (C-12) and methine carbon at δ_C_ 47.9 (C-18) and proton at δ_H_ 2.04 (m, 1H, H-18) and 2.38 (d, 1H, *J* = 7.2 Hz, H-19) showed ^2^*J* correlation with methine carbon at δ_C_ 48.2 (C-19) and quaternary carbon at δ_C_ 150.9 (C-20), respectively. Based on these ^1^H and ^13^C-NMR spectral data of **1**, summarized in Table 1, and the comparison with the literature values of lupeol [24], **1** was identified as 28,28,30-trihydroxylupeol. Selected C-H correlations in **1** established by HMBC are shown in Figure 2. In the genus *Ficus*, lupeol was previously isolated from *F. carica* [25], *F. micricarpa* [26] and *F. benjamina* [9], while lupeol acetate was isolated from *F. microcarpa* [26]. This is the first report of a hydroxy derivative of lupeol in *Ficus* species.

**Table 1:** ^1^H- and ^13^C-NMR spectroscopic data (600 MHz, CDCl_3_) for triterpenoids **1**–**3**.

Positions	Compound 1		Compound 2		Compound 3	
	δ_C_	δ_H_ (*J* in Hz)	δ_C_	δ_H_ (*J* in Hz)	δ_C_	δ_H_ (*J* in Hz)
1	38.1	1.28 (d, 4H, *J* = 6.6)	29.8	0.94, (m, 2H)	36.1	1.41 (m, 1H)
						1.31 (m,1H)
2	27.3	1.68 (d, 4H, *J* = 3.0)	25.7	1.01 (m, 2H)	28.2	1.42 (m, 1H)
						1.32 (m, 1H)
3	80.1	3.39 (dd, 1H, *J* = 10.8, 4.8)	80.1	3.44 (1H, t, *J* = 18.0), 4.68 (OH)	36.7	1.42 (m, 1H)
						1.33 (m, 1H)
4	39.6	-	43.0	-	30.8	-
5	55.6	1.32 (d, 1H, *J* = 7.2)	151.0	-	59.6	1.34 (m, 1H)
6	17.7	1.28 (m, 4H, *J* = 6.6)	109.3	4.79 (t, 1H, *J* = 6.0)	18.6	1.65 (m, 2H)
7	34.1	1.28 (m, 4H)	31.9	1.77 (m, 2H)	37.4	1.46 (m, 1H)
8	40.9	-	50.3	1.48 (m, 1H)	40.1	-
9	50.3	0.88 (m, 10H)	48.2	-	51.9	1.56 (m, 1H)
10	37.3	-	39.9	-	36.7	-
11	21.0	1.61 (m, 4H)	23.7	1.34 (m, 2H)	20.6	1.54 (m, 1H)
						1.29 (m, 1H)
12	25.1	1.68 (m, 4H)	22.7	1.31 (m, 2H)	25.2	1.55 (m, 1H)
						1.29 (m, 1H)
13	37.9	1.34 (m, 1H)	42.8	-	37.7	1.24 (m, 1H)
14	42.8	-	40.9	-	42.9	-
15	27.4	1.26 (m, 4H)	34.5	1.57 (m, 2H)	39.7	1.49 (m, 1H)
						1.00 (m, 1H)
16	35.5	1.26 (m, 4H)	29.4	1.63 (m, 2H)	40.4	1.86 (m, 1H)
						1.25 (m,1H)
17	43.0	-	55.6	1.48 (m,1H)	59.6	-
18	47.9	2.04 (m, 1H)	27.4	1.03 (s, 2H)	46.5	1.68 (m, 1H)
19	48.2	2.38 (d, 1H, *J* = 7.2)	17.7	1.04 s, 1H	37.2	2.09 (m, 1H)
20	150.9	-	35.3	1.68 (m, 1H)	150.9	-
21	29.7	1.88–1.94 (m, 2H)	95.1	4.80 (m, 1H)	29.2	1.60 (m, 6H)
22	39.9	1.34 (m, 4H)	35.5	1.18 (m, 2H)	23.0	1.75 (t, 2H, *J* = 5.4)
23	14.5	0.79 (s, 3H)	25.0	1.35 (m, 2H)	22.1	0.83 (s, 6H)
24	18.0	0.83 (s, 3H)	150.9	4.57 (m, 1H)	16.6	0.90 (s, 3H)
25	15.9	0.94 (s, 3H)	109.3	-	15.4	0.76 (s, 3H)
26	16.1	1.01(s, 3H)	69.2	4.03 (d, 1H, *J* = 12)	17.6	0.89 (s, 3H)
27	14.1	1.07 (s, 3H)	173.8	-	13.9	0.73 (s, 3H)
28	95.1	4.79 (t, 1H, *J* = 6 Hz)	14.1	0.80 (s, 3H)	183.5	-
29	109.3	4.69 (br s, 1H)	15.9	0.85 (s, 1H)	116.3	5.32 (brs, 1H)
		4.57 (br s, 1H)				
30	69.2	5.14 (m, 1H, H-30)	16.5	0.92 (q, 3H)	20.1	1.10 (s, 3H)
		5.19 (m, 1H, H-30)				

Compound **2** was isolated as a white powder with a melting point range of 150–155 °C. The IR spectrum displayed absorption bands for a carboxylic acid group (3215 cm^−1^, 1678 cm^−1^) and a keto function at 1735 cm^−1^ and unsaturation at 1643 cm^−1^. The HRESIMS showed a molecular ion at *m*/*z* 502.3290 [M]^+^, 479.1023 [M − 23]^+^, 453.0885 [M − CH_3_OH − 17]^+^ and 311.1563 [M − side chain (C_8_H_14_O_5_) − 1]^+^. Based on HRESIMS data its molecular formula was determined to be C_30_H_46_O_6_ and the molecular weight was established as 502.3290 [M]^+^. The ^1^H-NMR spectrum displayed characteristic signals for methyl protons at δ_H_ 0.80 (s, 3H, H-28), 0.85 (s, 1H, H-29), 0.92 (q, 3H, H-30) and 1.04 (s, 1H, H-19). One proton downfield signal appeared at δ_H_ 4.79 (t, 1H, *J* = 6.0 Hz, H-6) and was attributed to the vinylic H-6 proton. Another proton resonating as a triplet at δ_H_ 3.44 (1H, t, *J* = 18.0 Hz) was ascribed to a β-oriented H-3 carbinol proton. The ^13^C-NMR spectrum showed thirty carbon signals, including four methyls, ten methylenes, six methines and eight quaternary carbon signals. One methylene carbon at δ_C_ 69.2 and two methine carbons at δ_C_ 80.1 and δ_C_ 95.1 were typical for C-OH moieties. Important signals were observed for a carboxylic acid carbon at δ_C_ 173.8 (C-27), vinylic carbons at δ_C_ 151.0 (C-5) and δ_C_ 109.3 (C-6) carbinol carbons at δ_C_ 80.1 (C-3) and methyl carbons between δ_C_ 17.7–14.1. The ^1^H- and ^13^C-NMR data of **2** were compared to the reported data of other lanostanoic acids [22,27].

Proton and carbon connectivity established by HMQC and HMBC spectral analysis indicated that four methyl protons at δ_H_ 0.80, 0.80, 0.92 and 1.04 were connected to the C-28, C-29, C-30 and C-19 tertiary methyl protons, respectively, all attached to the saturated carbons. Two methyl groups resonating as a singlet were observed at δ_H_ 0.80 (s, 3H, H-28), 0.85 (s, 1H, H-29) and were attached to quaternary carbon at δ_C_ 43.0 (C-4). Methyl protons at δ_H_ 0.80 (s, 3H, H-28) and 0.85 (s, 1H, H-29) displayed ^3^*J* correlations with quaternary carbon at δ_C_ 151.0 (C-5) and the carbinol carbon at δ_C_ 80.1 (C-3). Meanwhile two methylene protons observed at δ_H_ 0.94, (m, 2H, H-1) and 1.01 (m, 2H, H-2) showed ^3^*J* and ^2^*J* correlations, respectively, with the same hydroxyl carbinol carbon at δ_C_ 80.1 (C-3). Another two methyl groups positioned at δ_H_ 1.04 (s, 1H, H-19) and 0.92 (q, 3H, H-30) were attached to quaternary carbons at δ_C_ 42.8 (C-13) and δ_C_ 40.9 (C-14), respectively. Three methylene proton signals at δ_H_ 1.34 (m, 2H, H-11), 1.31 (m, 2H, H-12) and 1.03 (s, 2H, H-18) were observed to show ^2^*J*, ^3^*J* and ^2^*J* correlations, respectively, with the quaternary carbon at δ_C_ 48.2 (C-9). One carbinol proton appearing as multiplet at δ_H_ 4.80 was attributed to C-21 and showed a ^2^*J* correlation with the methine carbon at δ_C_ 35.3 (C-20). The COSY spectrum revealed the presence of two sets of methyl groups at δ_H_ 0.80 (s, 3H, H-28) and δ_H_ 0.85 (s, 1H, H-29) coupled with each other and attached to a quaternary carbon at C-4 (δ_C_ 43.0) and another set of methyl protons at δ_H_ 0.92 (q, 3H, H-30) coupled with a methylene proton at δ_H_ 1.57 (m, 2H, H-15). The ^1^H- and ^13^C-NMR spectral data are summarized in Table 1. The C-H correlations established by HMBC are shown in Figure 2. Based on these evidences and comparisons with the spectral data of lanostene type triterpenoic acids [22,27], the structure of **2** was identified as 3,21,21, 26-tetrahydroxylanostanoic acid.

Compound **3** was isolated as a white powder with a melting point range of 285–290 °C. The UV spectrum showed an absorption at ν_max_ 254 nm. The IR spectrum displayed absorption bands for carboxylic acid hydroxyl groups at ν_max_ 2924 cm^−1^ (C-OH, acid), carboxylic acid carbonyl carbon at 1732 cm^−1^ (C=O, acid) and olefins at 1648 (C=C). There is no absorption band for an alcoholic hydroxyl (-OH, alcohol). The ESIMS (positive mode) showed a molecular ion at *m*/*z* 441.3663 [M + H]^+^ and 463.3474 [M + Na]^+^ while ESIMS (negative mode) showed a molecular ion at *m*/*z* 439.3759 [M − H]^–^ and 879.7791 [2M − H]^−^. HRESIMS (positive mode) displayed a molecular ion at *m*/*z* 457.2734 [M + OH]^+^. Its molecular formula was thus deduced to be C_30_H_48_O_2_ (calculated 440.3650 [M^+^] for C_30_H_48_O_2_). The ^1^H-NMR spectrum of **3** revealed signals for five tertiary methyls at δ_H_ 0.83 (s, 6H, H-23), 0.90 (s, 3H, H-24), 0.76 (s, 3H, H-25), 0.89 (s, 3H, H-26), 0.73 (s, 6H, H-27), a vinyl methyl at δ_H_ 1.10 ppm (s, 3H, H-30) and an *exo*-methylene proton at δ_H_ 5.32 ppm (brs, 1H, H-29).

The ^13^C-NMR spectrum showed six methyl groups at δ_C_ 22.1 (C-23), 16.6 (C-24), 15.4 (C-25), 17.6 (C-26), 13.9 (C-27), and 20.1 ppm (C-30), an *exo-*methylene group at δ_C_ 150.9 (C-20) and 116.3 (C-29) and a carboxylic acid group at δ_C_ 183.5 (C-28). In addition the ^13^C-NMR spectrum showed twelve methylene carbons, five methine carbons and six quaternary carbon signals. The ^1^H- and ^13^C-NMR data of **3** were very similar to those reported for betulinic acid [23]. The difference lies only the presence of a hydrogen instead a hydroxyl at C-3. These NMR spectral data indicated that compound **3** is a pentacyclic lupeol-type triterpenoic acid without a secondary hydroxyl-bearing carbinol carbon at the C-3 position. Proton and carbon connectivity was determined by HMQC and HMBC spectral analyses. Two methyl protons resonating as a singlet observed at δ_H_ 0.90 (H-24) and 0.83 (H-23) were attached to C-4 (δ_C_ 30.8) and correlated with carbons at δ_C_ 36.7 (C-3) and 59.6 (C-5), respectively, by a ^3^*J* correlation. One of these methyl protons also showed a ^2^*J* correlation with the quaternary carbon at C-4 (δ_C_ 30.8). Another methyl group at δ_H_ 0.76 (H-25) displayed ^3^J correlations to a methylene carbon at δ_C_ 36.1 (C-1) and two methyne carbons at δ_C_ 59.6 (C-5) and 51.9 (C-9). An *exo*-methylene proton at δ_H_ 5.32 (H-29) resonating as a broad singlet attached to a quaternary carbon at δ_C_ 150.9 (C-20) was correlated with a methine carbon at δ_C_ 37.2 (C-19). Two methyl groups at δ_H_ 0.73 (s, 3H, H-27) and 1.10 (s, 3H, H-30) resonating as a singlet were attached to a quaternary carbon at δ_C_ 42.9 (C-14) and 150.9 (C-20), respectively, whereas a methyl proton at δ_H_ 0.73 (s, 3H, H-27) showed ^3^*J* correlations with the methylene carbon at δ_C_ 39.7 (C-15), methine carbon δ_C_ 37.7 (C-13) and the proton at δ_H_ 1.10 (s, 3H, H-30) showed a ^2^*J* correlation with quaternary carbon at δ_C_ 150.9 (C-20). Two methylene protons at δ_H_ 1.86 (m, 1H, 16a) and 1.25 (m, 1H, 16b) displayed ^2^*J* correlations with δ_C_ 39.7 (C-15) and δ_C_ 59.6 (C-17). These methylene protons also showed ^3^*J* correlations with the methine carbon at δ_C_ 46.5 (C-18) and the quaternary carboxylic carbon at δ_C_ 183.5 (C-28). Similarly the carboxylic acid carbon at δ_C_ 183.5 (C-28) was correlated with methylene proton at δ_H_ 1.75 (t, 2H, *J* = 5.4 Hz, H-22) by ^3^*J* correlation. Two methine protons at δ_H_ 1.68 (m, 1H, H-18) and 2.09 (m, 1H, H-19) were observed to show ^3^*J* and ^2^*J* correlations with the quaternary carbon at δ_C_ 150.9 (C-20) and the proton at δ_H_ 2.09 (m, 1H, H-19) also showed a ^2^*J* correlation with δ_C_ 46.5 (C-18). In the same way the methine proton at δ_H_ 1.24 (m, 1H, H-13) was correlated with the methine carbon at δ_C_ 46.5 (C-18) and the quaternary carbon at δ_C_ 40.1 (C-8) by ^2^*J* and ^3^*J* correlations, respectively. Another methine proton at δ_H_ 1.56 (m, 1H, H-9) showed ^2^*J* and ^3^*J* correlations with the quaternary carbon at δ_C_ 36.7 (C-10) and 42.9 (C-14), respectively.

The COSY spectrum revealed the presence of one set of methylene protons at δ_H_ 5.32 (brs, 1H, H-29) attached to C-29 (δ_C_ 116.3) were coupled to each other and with one set of methyl proton δ_H_ 1.10 (s, 3H, H-30). Based on the above ^1^H-NMR, ^13^C-NMR information as well as the HMBC, HSQC and COSY data and comparison with the literature values of betulinic acid [23], the structure of **3** was identified as dehydroxybetulinic acid. The ^1^H- and ^13^C-NMR spectral data of **3** are summarized in Table 1. The positions of carbons and protons were established by 2D-NMR data (HMBC, HSQC and COSY) and the C-H correlations established by HMBC are shown in Figure 2. In the genus Ficus, betulinic acid and acetylbetulinic acid and betulonic acid were previously isolated from *F. microcarpa* [28]. This is the first report of dehydroxybetulinic acid from a *Ficus* species.

### 2.2. Chemotactic Activity

**Figure 3:**
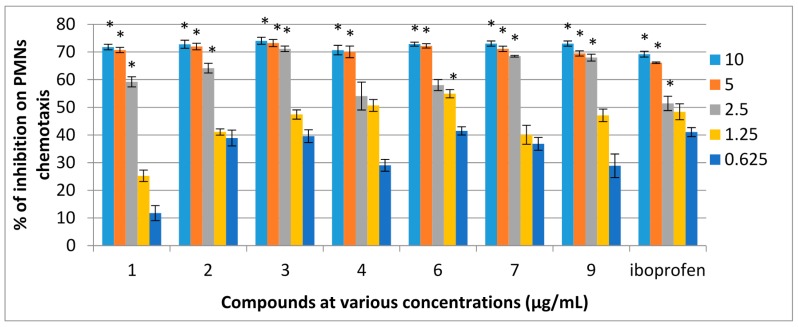
Dose dependent percentage of inhibition of compounds of *F. aurantiaca* on PMNs chemotaxis. Data are mean ± SEM (*n* = 3). * *p* < 0.05 is significant difference compared with the respective control determined by one-way ANOVA followed by Tukey’s test.

The cell viability test was performed using trypan blue to determine the toxicity of the compounds on immune cells at different concentrations. The elevated cell viability indicated that the compounds were nontoxic to immune cells and were able to modulate the cellular immune response of phagocytes. Cells were viable (>92%) at the concentrations of 6.25 and 100 μg/mL of the extracts after incubation for 2 h. All the compounds were tested for chemotaxis activity at five different concentrations (10, 5, 2.5, 1.25 and 0.625 μg/mL). Distance migrated by cell was specified in μm. The percentage of inhibition and IC_50_ values were calculated comparing the distance travelled by negative control and samples. The average distance travelled by the negative control (DMSO and HBSS, 1:1 ratio) was 15.8 μm while that for the positive control, ibuprofen, was 4.8 μm. Ibuprofen was used as a positive control as it was found to be the most effective drug in a study to determine the effect of selected nonsteroidal anti-inflammatory drug (NSAIDs) in blocking the migration of PMNs [29,30]. The results showed that DMSO did not affect the movement of cells. Seven compounds showed high percentage of inhibition (70% to 80%), significantly different to the control (*p* < 0.05). Among the ten compounds, seven showed strong inhibitory activities with dose-dependent effects on the migration of PMNs towards the chemo attractant (fMLP). Compounds **1**–**4**, **6**, **7** and **9** exhibited strong (more than 70%) inhibition and the IC_50_ values of these compounds were comparable to that of ibuprofen. The percentage of inhibitions of PMNs chemotaxis for the active compounds is shown in Figure 3. The IC_50_ values of active compounds with positive control are shown in Table 2.

**Table 2:** IC_50_ values of compounds isolated from *F. aurantiaca*.

Compounds	IC_50_ Value (μM)		
	ROS		Chemotaxis
	PMNs	WB	
28,28,30-Trihydroxylupeol (**1**)	9.2 ± 0.5	11.5 ± 0.05	6.8 ± 0.1
3,21,21,26-Tetrahydroxylanostanoic acid (**2**)	0.9 ± 0.03	0.1 ± 0.3	2.8 ± 0.1
Dehydroxybetulinic acid (**3**)	0.9 ± 0.1	1.7 ± 0.1	2.5 ± 0.1
Taraxerone (**4**)	1.3 ± 0.1	4.6 ± 0.08	4.1 ± 0.5
Taraxerol (**5**)	-	-	-
Ethyl palmitate (**6**)	1.1 ± 0.05	0.7 ± 0.05	3.7 ± 0.2
Herniarin (**7**)	0.5 ± 0.5	1.6 ± 0.1	8.2 ± 0.2
Stigmasterol (**8**)	-	-	-
Ursolic acid (**9**)	0.8 ± 0.5	1.3 ± 0.8	3.6 ± 0.2
Acetylursolic acid (**10**)	-	-	-
Aspirin (positive control)	9.4 ± 0.5	11.6 ± 0.3	-
Ibuprofen (positive control)	-	-	6.7 ± 0.5

### 2.3. Reactive Oxygen Species (ROS) Inhibitory Activity of Isolated Pure Compounds on Human Whole Blood (WB) and PMNs

**Figure 4:**
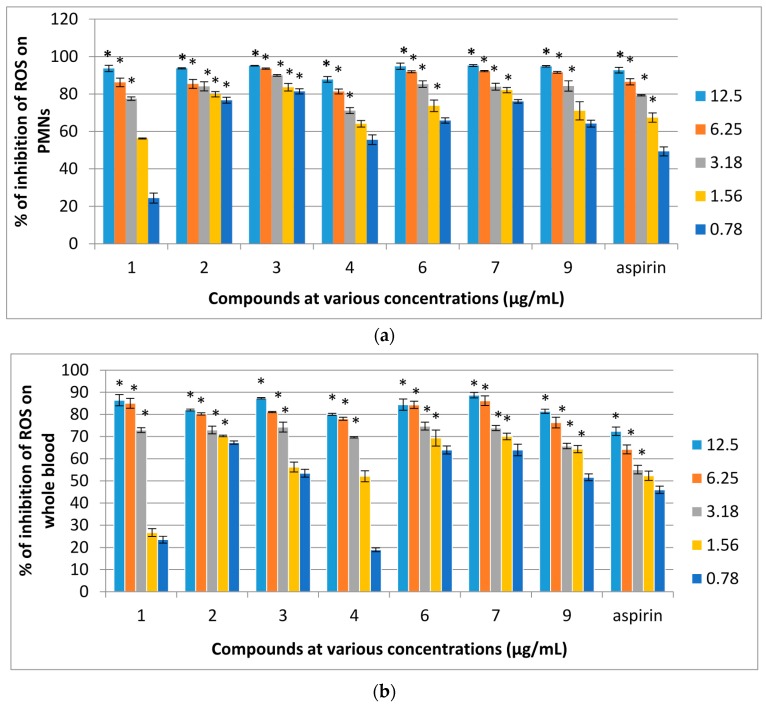
(**a**) Dose dependent percentage of inhibition of ROS inhibitory activity of compounds isolated from *F. aurantiaca* on PMNs and (**b**) whole blood assayed by luminol amplified chemiluminescence. Data are mean ± SEM (*n* = 3).* *p* < 0.05 is significant difference compared with the respective control determined by one-way ANOVA followed by Tukey’s test.

Compounds **1**–**10** were evaluated for their effects on the oxidative burst of PMNs. Of the ten compounds, **1**–**4**, **6**, **7** and **9** were strongly active against PMNs. The IC_50_ values of these compounds were lower than that of aspirin which was used as a positive control. The result showed that the compounds inhibited ROS generation during the metabolic phase of phagocytises in a dose-dependent manner as the concentrations of samples increased the percentage of inhibition was also increased. Further evaluation of compounds **1**–**10** for their effects on oxidative burst on human whole blood showed that compounds **1**–**4**, **6**, **7** and **9** exhibited remarkable activity for luminol-enhanced chemiluminescence. In addition, compounds **1**–**3**, **6**, **7** and **9** showed strong inhibition on both PMNs and whole blood whereas inhibition of PMNs was higher than that of whole blood. The dose dependent ROS inhibitory effects on the oxidative burst of human whole blood (b) and PMNs (a) for active compounds are shown in Figure 4 and their IC_50_ values are shown in Table 2.

The potency of triterpenoids from *F. aurantiaca* as inhibitors of chemotaxis and ROS production of neutrophils is in agreement with many previous studies. Triterpenoids, especially pentacyclic triterpenes like compounds **1** and **3** were implicated in studies on the mechanisms of action and pharmacological effects of many medicinal plants used in folk medicine against diseases related to the immune system such as anti-inflammatory, antiviral, antimicrobial, antitumoral agents, as well as immunomodulating effects. Several of them are implicated in the resolution of immune diseases, although their effects have not always been clearly correlated [31]. Oleanolic acid and its related compounds such as ursolic acid and betulinic acid are known to be anti-inflammatory and immuno-suppressive through their reduction of relevant cytokines such as interleukin-1 (IL-1) interleukin-6 (IL-6), and tumor necrosis factor-α (TNF-α), as well as their effect on the classic pathway of complement activation though the inhibition of C3 convertase. Triterpenoic acid such as oleanolic acid and 3-*epi*-katonic acid also inhibited adenosine deaminase, an enzyme which is found at increased levels in various immune diseases [31,32]. The immunosuppressive effects of *Tripterygium wilfordii* might also involve triterpenoids, as well as compounds such as oleanolic acid, 3-*epi*-katonic acid, terpenoic acid. Eight cycloartanes isolated from *Astragalus melanophrurius* (Fabaceae) were found to show interesting immunomodulatory activity in an isolated human lymphocyte stimulation test [32]. The effects of a mixture of triterpenes from *Quillaja saponaria* (Rosaceae) on the production of IL-1 and IL-6, as well as their role in the activation of antigen-presenting cells (APC), a prerequisite for the development of immune responses have been reported [33].

Triterpenes of diverse structural types are widely distributed in prokaryotes and eukaryotes. The physiological function of these compounds is generally supposed to be a chemical defense against pathogens. Triterpenes act against certain pathogens causing human and animal diseases, such as imflammation. This may be primarily due to the hydrophobic nature of most of the compounds [34]. In this study, among the eight triterpenes, there are two lupine-type skeletons (compounds **1** and **3**), two taraxerane-type (**4** and **5**), two ursane-type (**9** and **10**), one lanostane-type skeleton (**2**) and one stigmastane skeleton (**8**). A variety of terpenoids with oleanane-, ursane-, taraxerane-, lupine- and friedelane-type skeletons previously identified in many higher plants can act as precursors for many biomarkers found in biological screening. Oleanane, ursane and lupane triterpenes with carboxylic acid groups and alcoholic derivatives of oleanane, ursane and lupane are active against inflammation. However, it is difficult to identify exact molecular motifs, largely spread among these terpenes and implicated in their anti-inflammatory action. Some of the terpenoids act as plant hormones regulating different physiological roles, but some secondary metabolites is concerned in host defence and in the protection of the plant or animal from possible pathogens. Recent research in the field of the regulation of innate immunity from insects to mammals has established the existence of a previously unexpected protection in the pathways (receptors, kinases and effector molecules) that are involved in this process [35]. We suggest that the general analysis of the relationship between chemical structure and immunomodulatory activity of the isolated triterpenes may be related to the presence of an oxygenated group at C-3 and carboxyl group at C-28.

## 3. Experimental Section

### 3.1. General Information

Melting points were determined with an electrothermal apparatus (digital series) and were uncorrected. Ultraviolet (UV) spectra were recorded with a Shimadzu UV-1601 Spectrophotometer (Shimadzu Corp., Tokyo, Japan), for ethanol and methanol solutions in 1 cm Quartz cells. IR spectra were recorded on a GX FTIR instrument (Perkin-Elmer Corp., Waltham, MA, USA) using potassium bromide pellets and sodium chloride cells. NMR spectral analyses were carried out with a FT-NMR 600 MHz Cryoprobe spectrometer (Bruker, Basel, Switzerland). ^1^H-^1^H COSY, ^1^H-^13^C HSQC and ^1^H-^13^C HMBC were obtained with the usual pulse sequence and data processing was performed with the ACD lab software (Version 12, Advanced Chemistry Development, Toronto, ON, Canada). Mass spectra ESIMS and HRESIMS were measured on Micro TOF-Q mass spectrometer (Bruker, Basel, Switzerland). All experiments were carried out at Universiti Kebangsaan Malaysia (UKM). Analytical Thin Layer Chromatography (TLC) was carried out on pre-coated silica gel 60 GF 254 (20 × 20) TLC plate (Merck, art 5554, Darmstadt, Germany). The plates were visualized under ultraviolet light (λ_254_ nm, model UVGL-58) and by charring the compounds after spraying the plates with 10% sulphuric acid or Dragendorff reagent. Vacuum liquid chromatography was carried out over silica gel type H (10–0 μm, Sigma Chemical Co., St. Louis, MO, USA). The conventional column chromatography was done by using silica gel 60 (230–400 mesh ASTM, Merck, art 9385) or Sephadex LH20 (GE Healthcare Bio-Sciences AB, Uppsala, Sweden). Radial chromatography (centrifugal chromatography) was performed on a Chromatotron (Analtech Inc., Newark, Denmark) using silica gel 60 GF 254 containing gypsum (TLC grade) (Merck, art. 7749). Organic solvents such as hexane, ethyl acetate, chloroform, diethyl ether and methanol were purchased from Merck.

### 3.2. Plant Material

*Ficus aurantiaca* Griff was collected from Banting, Selangor, Malaysia and a voucher specimen (SM2109) was identified and deposited at the Herbarium of Universiti Kebangsaan Malaysia (UKM), Bangi, Malaysia.

### 3.3. Extraction and Isolation

Extraction and isolation of compounds from *F. aurantiaca* was carried out by modification of the method described by Rukachaisirikul [36]. The plant material was dried at room temperature and its air-dried stems were ground. The ground powder (196 g) was sequentially extracted thrice with *n*-hexane, ethyl acetate and methanol (500 mL each) by soaking at least for 48 h at room temperature for each time. After filtration each of the solvent extracts was evaporated to dryness under reduced pressure using a rotary evaporator to yield crude n-hexane (7.8 g), ethyl acetate (4.0 g) and methanol (10.0 g) extracts, respectively. The separation of hexane, ethyl acetate and methanol extracts was carried out using vacuum liquid chromatography (VLC), column chromatography and Chromatotron over silica gel, respectively.

The *n*-hexane crude extract (6.0 g) of *F. aurantiaca* was chromatographed using VLC on silica gel 60 GF_254_ (TLC grade) and hexane–ethyl acetate (10:0 to 0:10, *v*/*v*) as solvent system to give sixteen fractions (F1–F16) that were analyzed using TLC and combined into five fractions (Fa1–Fa5) based on their TLC profiles. For further isolation and purification, each fraction was subjected to column chromatography (CC) using silica gel 60 (230–400 mesh ASTM). Fa1 (1.234 g) was subjected to CC on silica gel as stationary phase and hexane–ethyl acetate (10:0 to 0:10, *v*/*v*) of increasing polarity as mobile phase to obtain seven subfractions (Fa1A–Fa1G). Subfractions Fa1A and Fa1B were subjected to silica gel CC eluted with hexane–ethyl acetate (9:1 to 5:5 *v*/*v*) and further recrystallized from 100% *n*-hexane to obtain compound **6**. Fraction Fa3 (2.135 g) was purified by silica gel CC eluted with hexane–ethyl acetate (9:1 to 0:10 *v*/*v*) to obtain compound **7**.

The ethyl acetate crude extract (3.0 g) of *F. aurantiaca* was subjected to silica gel CC eluted with hexane–ethyl acetate (5:5 to 0:10, *v*/*v*) and ethyl acetate–methanol (10:0 to 6:4, *v*/*v*) to obtain eighteen fractions (F1–F18). Based on the TLC profiles, these eighteen fractions were combined into six fractions (Faa–Faf). Fractions Faa (322.1 mg) and Fab (173.2 mg) were separated and purified using repeated CC to obtain compounds **1** and **4**, respectively. In the same way **5**, **2** and **8** were obtained from fractions Fad (321.7 mg), Fae (523.2 mg) and Faf (457.2 mg), respectively. Similarly the methanol extract of *F. aurantiaca* (8.0 g) was fractionated using VLC on silica gel 60 GF_254_ using ethyl acetate-methanol of increasing polarity as eluent and the fractions were purified by silica gel CC to obtain subfractions FF1–FF10. Subfractions FF2 (567.0 mg), FF5 (234.0 mg) and FF7 (638.0 mg) was run on a Chromatotron eluted with ethyl acetate–methanol (10:0 to 2:8, *v*/*v*) to yield compounds **9**, **3** and **10**, respectively. To summarize, compounds **6** and **7** were obtained from the *n-*hexane extract; compounds **1**, **4**, **5**, **2** and **8** were isolated from ethyl acetate extract while compounds **9**, **3** and **10** were isolated from the methanol extract. The structure elucidation of the isolated compounds was performed using UV, IR, ESIMS and NMR spectral data analyses and comparison with appropriate literature values.

*28,28,30-Trihydroxylupeol* (**1**): White powder (20.0 mg), melting point 222–224 °C, UV λ_max_ (C_2_H_5_OH) nm: 272, 210 (log ε 3.4, 2.2). IR λ_max_ (CHCl_3_) cm^−1^: 3310, 2920, 2158, 2042, 1976, 1611, 1411, 1021. ^1^H-NMR (CDCl_3_): δ_H_ 0.79 (s, 3H, H-23), 0.83 (s, 3H, H-24), 0.94 (s, 3H, H-25), 1.01 (s, 3H, H-26), 1.07 (s, 3H, H-27), 0.88 (m, 10H, H-9), 1.26 (m, 4H, H-15, H-16), 1.28 (m, 4H, *J* = 6.6 Hz, H-6, H-7), 1.32 (m, 1H, *J* = 7.2 Hz, H-5), 1.34 (m, 4H, H-22, H-13), 1.61 (m, 4H, H-11), 1.68 (m, 4H, *J* = 3.0 Hz, H-2, H-12), 1.88–1.94 (m, 2H, H-21), 2.04 (m, 1H, H-18), 2.38 (d, 1H, *J* = 7.2 Hz, H-19), 3.39 (dd, 1H, *J* = 10.8, 4.8 Hz, H-3), 4.57 (br s, 1H, H-29), 4.69 (br s, 1H, H-29), 4.79 (t, 1H, *J* = 6.0 Hz, H-28), 5.14 (m, 1H, H-30) 5.19 (m, 1H, H-30). ^13^C-NMR (CDCl_3_): δ_C_ 14.1(C-27), 14.5 (C-23), 15.9 (C-25), 16.1 (C-26), 17.7 (C-6), 18.0 (C-24), 21.0 (C-11), 25.1 (C-12), 27.3 (C-2), 27.4 (C-15), 29.7 (C-21), 34.1 (C-7), 35.5 (C-16), 37.3 (C-10), 37.9 (C-13), 38.1 (C-1), 39.6 (C-4), 39.9 (C-22), 40.9 (C-8), 42.8 (C-14), 43.0 (C-17), 47.9 (C-18), 48.2 (C-19), 50.3 (C-9), 55.6 (C-5), 69.2 (C-30), 80.1(C-3), 95.1 (C-28), 109.3 (C-29), 150.9 (C-20). ESIMS *m*/*z*: 463.3393 [M + H − 12]^+^, 931.7436 [2M + H − 18]^+^, HRESIMS *m*/*z*: 474.1465 [M]^+^, calculated 474.1465 for molecular formula C_30_H_50_O_4_.

*3,21,21,26-Tetrahydroxylanostanoic acid* (**2**): White powder (17.2 mg), melting point 150–155 *°*C, UV ν_max_ (C_2_H_5_OH) nm: 272, 207 (log ε 2.3, 2.8). IR ν_max_ (CHCl_3_) cm^−1^: 3215, 2915, 2850, 2134, 1985, 1735, 1678, 1642, 1472, 1120. ESIMS (negative mode) *m*/*z*: 502.3290 [M]^+^, 479.1023 [M − 23]^+^, 453.0885 [M − CH_3_OH − 17]^+,^ and 311.1563 [M − side chain (C_8_H_14_O_5_) − 1]^+^, HRESIMS (positive mode) *m*/*z*: 503.3570 [M + H]^+^, calculated 502.3290 for molecular formula C_30_H_50_O. ^1^H-NMR (CDCl*_3_*): δ_H_ 0.80 (s, 3H, H-28), 0.85 (s, 1H, H-29), 0.92 (q, 3H, H-30), 0.94 (m, 2H), 0.99 (s, 2H, H-18), 1.01(m, 2H, H-2), 1.03 (s, 1H, H-19), 1.18 (m, 2H, H-22), 1.31 (m, 2H, H-12), 1.34 (m, 2H, H-11), 1.35 (m, 2H, H-23), 1.48 (m, 1H, H-8, H-17), 1.57 (m, 2H, H-15), 1.63 (m, 2H, H-16), 1.68 (m, 1H, H-20), 1.77 (m, 2H, H-7), 3.44 (t, 1H, H-3), 4.03 (d, 1H, *J* = 12.0 Hz, H-26), 4.57 (m,1H, H-24), 4.79 (t, 1H, *J* = 6.0 Hz, H-6), 4.80 (m, 1H, H-21), 2.25 (C-21), 2.30 (C-21), 4.68 (C-3), 4.69 (C-26). ^13^C-NMR (CDCl*_3_*): δ_C_ 29.8 (C-1), 25.7 (C-2), 80.1 (C-3), 43.0 (C-4), 151.0 (C-5), 109.3 (C-6), 31.9 (C-7), 50.3 (C-8), 48.2 (C-9), 39.9 (C-10), 23.7 (C-11), 22.7 (C-12), 42.8 (C-13), 40.9 (C-14), 34.5 (C-15), 29.4 (C-16), 55.6 (C-17), 27.4 (C-18), 17.7 (C-19), 35.3 (C-20), 95.1 (C-21), 35.5 (C-22), 25.0 (C-23), 150.9 (C-24), 109.3 (C-25), 69.2 (C-26), 173.8 (C-27), 14.1 (C-28), 15.9 (C-29), 16.5 (C-30).

*Dehydroxybetulinic acid* (**3**): White powder (17.4 mg) with a melting point 285–290 °C. UV λ_max_ (C_2_H_5_OH) nm: 195. IR ν_max_(CHCl_3_) cm^−1^: 2924, 2238, 2158, 1732 1648, 1463, 1248 and 971 cm^−1^. ESIMS (positive mode) *m*/*z*: 441.3663 [M + H]^+^ 463.3474 [M + Na]^+^and ESIMS (negative mode) *m*/*z*: 439.3759 [M − H]^−^, 879.7791 [2M − H]. HRESIMS (positive mode) *m*/*z*: 457.2734 [M + OH]^+^, calculated 440.3787 for molecular formula C_30_H_48_O_2_. ^1^H-NMR (CDCl_3_): δ_H_ 1.41 (m, 1H, H-1), 1.31 (m, 1H, H-1), 1.42 (m, 1H, H-2), 1.32 (m, 1H, H-2), 1.42 (m, 1H, H-3), 1.33 (m, 1H, H-3), 1.34 (m, 1H, H-5), 1.65 (m, 2H, H-6), 1.46 (m, 1H, H-7), 1.56 (m, 1H, H-9), 1.54 (m, 1H, H-11), 1.29 (m, 1H, H-11), 1.55 (m, 1H, H-12), 1.29 (m, 1H, H-12), 1.24 (m, 1H, H-13), 1.49 (m, 1H, H-15), 1.00 (m, 1H, H-15), 1.80 (m, 1H, H-16), 1.25 (m, 1H, H-16), 1.68 (m, 1H, H-18), 2.09 (m, 1H, H-19), 1.60 (m, 6H, H-21), 1.75 (t, 2H, *J* = 5.4 Hz, H-22), 0.83 (s, 6H, H-23), 0.90 (s, 3H, H-24), 0.76 (s, 3H, H-25), 0.89 (s, 3H, H-26), 0.73 (s, 6H, H-27), 5.32 (br s, 1H, H-29), 1.10 (s, 3H, H-30). ^13^C-NMR (CDCl_3_): δ_C_ 36.1 (C-1), 28.2 (C-2), 36.7 (C-3), 30.8 (C-4), 59.6 (C-5), 18.6 (C-6), 37.4 (C-7), 40.1 (C-8), 51.9 (C-9), 37.7 (C-10), 20.6 (C-11), 25.2 (C-12), 37.7 (C-13), 42.9 (C-14), 39.7 (C-15), 40.4 (C-16), 59.6 (C-17), 46.5 (C-18), 37.2 (C-19), 150.9 (C-20), 29.2 (C-21), 23.0 (C-22), 22.1 (C-23), 16.6 (C-24), 15.4 (C-25), 17.6 (C-26), 13.9 (C-27), 183.5 (C-28), 116.3 (C-29), 20.1 (C-30).

### 3.4. Chemicals, Reagents and Equipment

Serum opsonized zymosan A (*Saccharomyses cerevisiae* suspensions and serum), luminol (3-aminophthalhydrazide), phosphate buffer saline (PBS), Hanks Balance Salt Solution (HBSS^++^), ficoll, *N*-formyl-methionylleucyl-phenilalanine (fMLP), tryphan blue, phorbol 12-myristate 13-acetate (PMA), dimethyl sulfoxide (DMSO), acetylsalicylic acid (purity 99%) and ibuprofen (purity 99%) were purchased from Sigma. Haematoxylin and xylene were obtained from BDH (London, UK). Chemiluminscence measurements were carried out on a Luminoscan Ascent luminometer (Thermo Scientific, Loughborough, UK). A Boyden chamber with a 3 and 5 μm polycarbonate membrane filter separating the upper and lower compartments was purchased from Neuro probe (Cabin John, Montgomery County, MD, USA).

### 3.5. Isolation of Polymorphonnuclear Leucocytes (PMNs)

Human blood was collected from a healthy volunteer fasted for at least 8 h. PMNs were isolated by Ficol-gradient separation following the published method [37]. The use of human blood in this study was approved by the Human Ethics Committee, Universiti Kebangsaan Malaysia Medical Centre (HUKM), Cheras with permission (number FF-220-2008). Cell counts were performed using a haemocytometer.

### 3.6. Cell Viability

Cell viability tests were performed using trypan blue dye exclusion method with the modification of a published procedure [38]. The neutrophils (1 × 10^6^ mL^−1^) were incubated with 6.25 or 100 μg/mL of pure compounds in triplicate at 37 °C for 1 to 2 h. The cell death was indicated by the blue dye uptake. The percentage of cell viability was considered from the total cell counts.

### 3.7. Chemiluminescence Assay

Luminol enhanced chemiluminescence assay was carried out using the modification of the published method [39]. In brief, 25 μL of human whole blood or 25 μL of isolated PMN cells were suspended in HBSS^++^ into each well of 96-well microplate. The plate was incubated with 25 μL of tested compounds and aspirin at five different concentrations (12.5, 6.25, 3.18, 1.56 and 0.78 μg/mL) of each sample for 50 min at 37 °C in a luminoscan while 25 μL of HBSS^++^ was used as the negative control. The cells were induced with 25 μL of serum opsonized zymosan (SOZ) followed by 25 μL of luminol into each well. Then HBSS^++^ solution was added into each well to make the final volume of 200 μL. Aspirin was used as a positive control while the negative control contained zymosan, luminol, DMSO (0.5%), HBSS^++^ and cells. The percentage of inhibition was calculated by the measurement of RLU (reading luminometer unit) of peak and total integral values with repeated scans.

### 3.8. Chemotaxis Assay

The assay was carried out using the modified 48-well Boyden chamber method [40]. The Boyden chamber contains 48 wells with a diameter of 8 μm and is divided into two compartments by a filter separation. In brief, 25 μL of chemo attractant, fMLP (10^−8^ M, diluted with chemo attractant buffer solution) was added to the lower compartment of the Boyden chamber. PMN cell suspension (45 μL) with 5 μL of test compounds and ibuprofen at five different concentrations (6.25, 12.5, 25, 50, and 100 μg/mL) were added to the upper compartment of the chamber whereas the negative control contained 45 μL of PMN cell suspension and 5 μL of chemo attractant buffer. Ibuprofen was used as a positive control. The final concentrations of test compounds and ibuprofen in the wells were 0.625, 1.25, 2.5, 5 and 10 μg/mL. The chamber was incubated in 5% carbon dioxide incubator for 1 h at 37 °C. After incubation the polycarbonate membrane (where the migrated cells were remained) was stained with PBS, methanol (99.5%), haematoxylin, distilled water, ethanol (70% and 95.8%) and xylene respectively. The distance of cell’s migration was measured using a low power microscope with magnifications 40×.

### 3.9. Statistical Analysis

Data were analyzed using One-way ANOVA, *post hoc* Tukey’s test. *p* < 0.05 was considered to be statistically significant using the SPSS 17.0 (SPSS Inc., Chicago, IL, USA) statistical software. All values were characterized as mean ± SEM. Probit programme was used to determine the IC_50_ values for active compounds.

## 4. Conclusions

In conclusion, a phytochemical investigation of the stem of *F. aurantiaca* afforded three new triterpenoids—28,28,30-trihydroxylupeol (**1**), 3,21,21,26-tetrahydroxylanostanoic acid (**2**) and dehydroxybetulinic acid (**3**) together with five known triterpenoids: taraxerone (**4**), taraxerol (**5**), stigmasterol (**8**), ursolic acid (**9**), acetyl ursolic acid (**10**), one coumarin, herniarin (**7**) and one unsaturated hydrocarbon, ethyl palmitate (**6**). This is the first report on the phytochemical investigation and biological effects of compounds from *F. aurantiaca* on PMN chemotaxis and ROS inhibitory activity of human whole blood and PMNs. Compounds **1**–**3**, **6**, **7** and **9** showed the strongest inhibitory activities for both chemiluminescence and chemotaxis whereas the IC_50_ values were lower than those of the standard drugs used. This study provided evidence that *F. aurantiaca* is a source of new immunomodulatory compounds to modulate the innate immune response of phagocytes and further studies are needed to investigate the mechanisms of their immuno-modulatory responses.
